# Aqua­bis(2-amino-1,3-thia­zole-4-acetato-κ^2^ *O*,*N* ^3^)nickel(II)

**DOI:** 10.1107/S1600536809017978

**Published:** 2009-05-20

**Authors:** Qiu-Fen He, Dong-Sheng Li, Jun Zhao, Xi-Jun Ke, Cai Li

**Affiliations:** a College of Mechanical & Materials Engineering, Functional Materials Research Institute, China Three Gorges University, Yichang 443002, People’s Republic of China

## Abstract

In the crystal structure of the title compound, [Ni(C_5_H_5_N_2_O_2_S)_2_(H_2_O)], the Ni^II^ cation is located on a twofold rotation axis and chelated by two 2-amino-1,3-thia­zole-4-acetate (ata) anions in the basal coordination plane; a water mol­ecule located on the same twofold rotation axis completes the distorted square-pyramidal coordination geometry. Inter­molecular O—H⋯O and N—H⋯O hydrogen bonding, as well as π–π stacking between parallel thia­zole rings [centroid–centroid distance 3.531 (8) Å], helps to stabilize the crystal structure.

## Related literature

For general background to the potential use of discrete and polymeric metal-organic complexes as functional materials in catalysis, mol­ecular recognition, separation and non-linear optics, see: Batten & Robson (1998 ▶); Fujita *et al.* (1994 ▶); Han *et al.* (2008 ▶); Wu *et al.* (2001 ▶).
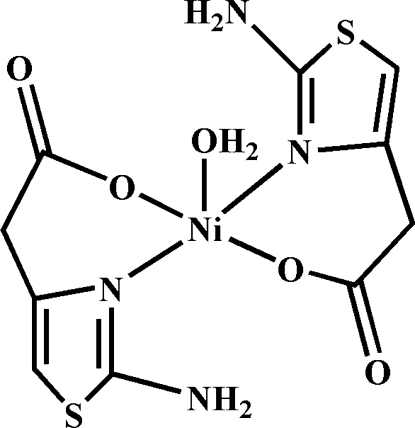


## Experimental

### 

#### Crystal data

[Ni(C_5_H_5_N_2_O_2_S)_2_(H_2_O)]
*M* *_r_* = 391.07
Monoclinic, 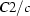

*a* = 12.0875 (12) Å
*b* = 9.1278 (9) Å
*c* = 12.7715 (12) Å
β = 95.1190 (10)°
*V* = 1403.5 (2) Å^3^
*Z* = 4
Mo *K*α radiation
μ = 1.71 mm^−1^
*T* = 293 K
0.12 × 0.10 × 0.06 mm

#### Data collection

Bruker SMART CCD diffractometer
Absorption correction: multi-scan (*SADABS*; Sheldrick, 1996 ▶) *T*_min_ = 0.821, *T*_max_ = 0.904
3487 measured reflections
1231 independent reflections
1119 reflections with *I* > 2σ(*I*)
*R*_int_ = 0.014

#### Refinement

*R*[*F* ^2^ > 2σ(*F* ^2^)] = 0.024
*wR*(*F* ^2^) = 0.065
*S* = 1.01
1231 reflections
102 parameters
H-atom parameters constrained
Δρ_max_ = 0.31 e Å^−3^
Δρ_min_ = −0.29 e Å^−3^

### 

Data collection: *SMART* (Bruker, 1997 ▶); cell refinement: *SAINT* (Bruker, 1997 ▶); data reduction: *SAINT*; program(s) used to solve structure: *SHELXTL* (Sheldrick, 2008 ▶); program(s) used to refine structure: *SHELXTL*; molecular graphics: *SHELXTL*; software used to prepare material for publication: *SHELXTL*.

## Supplementary Material

Crystal structure: contains datablocks I, global. DOI: 10.1107/S1600536809017978/xu2518sup1.cif

Structure factors: contains datablocks I. DOI: 10.1107/S1600536809017978/xu2518Isup2.hkl

Additional supplementary materials:  crystallographic information; 3D view; checkCIF report

## Figures and Tables

**Table 1:** Selected bond lengths (Å)

Ni1—O1	2.0243 (15)
Ni1—O3	1.999 (2)
Ni1—N2	2.0465 (18)

**Table 2:** Hydrogen-bond geometry (Å, °)

*D*—H⋯*A*	*D*—H	H⋯*A*	*D*⋯*A*	*D*—H⋯*A*
N1—H1*A*⋯O1^i^	0.86	2.10	2.816 (3)	140
N1—H1*B*⋯O2^ii^	0.86	1.99	2.839 (3)	170
O3—H3⋯O2^iii^	0.82	1.94	2.7211 (19)	158

Symmetry codes: (i) 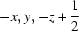; (ii) 
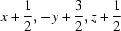
; (iii) 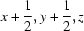.

**Fig. 1.**
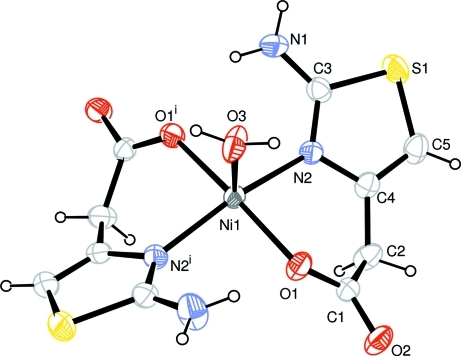
The molecular structure of the title compound with thermal ellipsoids plotted at 50% probability [symmetry code: (i) -x, y, -z + 1/2].

## supplementary crystallographic information

### Comment

The rational design and synthesis of novel discrete and polymeric metal-organic complexes have attracted intense interest owing to the realisation of their potential for use as functional materials in catalysis, molecular recognition, separation, and nonlinear optics (Batten & Robson, 1998; Fujita *et al.*, 1994). As for the construction of these inorganic/organic hybrid materials, carboxylate ligands have proven to be an efficacious choice (Wu *et al.*, 2001). The employment of multifunctional ligands bearing both anionic and neutral donor atoms, such as nicotinate, isonicotinate, and various pyridinedicarboxylates, has resulted in the preparation of many functional coordination polymers, some with intriguing optical or gas sorption properties (Han *et al.*, 2008). Herein we report the hydrothermal synthesis, structural characterization of the title complex.

The molecular structure of the title complex is shown in Fig. 1. The Ni^II^ ion is located on a twofold rotation axis and has a slightly distorted square-pyramidal geometry formed by two oxygen atoms, two nitrogen atoms from ata ligands and one coordinated water molecules (Table 1). The amido N atoms forms N—H···O hydrogen bonds with carboxylate O atoms, linking the molecules into one dimentional chains, which are then linked into a two-dimensional sheet by aromatic π-π stacking between S1-thiazole and S1^i^-thiazole [symmetry code: (i) -*x*, 1 - *y*, 1 - *z*] rings [centroid–centroid distance 3.531 (8) Å]. Furthermore, the two-dimensional layers are extended to a three-dimensional supramolecular structure by O—H···O hydrogen bonds (Table 2).

### Experimental

A mixture of Ni(CH_3_COOH)_2_.4H_2_O (0.025 g, 0.1 mmol), 2-amino-4-thiazoleacetic acid (0.0316 g, 0.2 mmol) and distilled water (10 ml) was sealed in a 25 ml Teflon-lined stainless autoclave. The pH value of the mixture was adjusted to 6 by a aqueous solution of NaOH (0.1 mol/*L*), and then heated at 393 K for 3 d. Green crystals were obtained on cooling to room temperature.

### Refinement

H atoms were placed in calculated positions and treated using a riding-model approximation with C—H = 0.93, with *U*_iso_(H) = 1.2*U*_eq_(C); O—H = 0.82 and N—H = 0.86 Å, *U*_iso_(H)=1.5*U*_eq_(O,*N*).

### Crystal data

**Table:**

[Ni(C_5_H_5_N_2_O_2_S)_2_(H_2_O)]	*F*(000) = 800
*M**_r_* = 391.07	*D*_x_ = 1.851 Mg m^−^^3^
Monoclinic, *C*2/*c*	Mo *K*α radiation, λ = 0.71073 Å
Hall symbol: -C 2yc	Cell parameters from 2106 reflections
*a* = 12.0875 (12) Å	θ = 2.8–25.0°
*b* = 9.1278 (9) Å	µ = 1.71 mm^−^^1^
*c* = 12.7715 (12) Å	*T* = 293 K
β = 95.119 (1)°	Prism, green
*V* = 1403.5 (2) Å^3^	0.12 × 0.10 × 0.06 mm
*Z* = 4	

### Data collection

**Table:**

Bruker SMART CCD diffractometer	1231 independent reflections
Radiation source: fine-focus sealed tube	1119 reflections with *I* > 2σ(*I*)
graphite	*R*_int_ = 0.014
CCD Profile fitting scans	θ_max_ = 25.0°, θ_min_ = 2.8°
Absorption correction: multi-scan (*SADABS*; Sheldrick, 1996)	*h* = −14→14
*T*_min_ = 0.821, *T*_max_ = 0.904	*k* = −5→10
3487 measured reflections	*l* = −15→14

### Refinement

**Table:**

Refinement on *F*^2^	Primary atom site location: structure-invariant direct methods
Least-squares matrix: full	Secondary atom site location: difference Fourier map
*R*[*F*^2^ > 2σ(*F*^2^)] = 0.024	Hydrogen site location: inferred from neighbouring sites
*wR*(*F*^2^) = 0.065	H-atom parameters constrained
*S* = 1.01	*w* = 1/[σ^2^(*F*_o_^2^) + (0.034*P*)^2^ + 2.301*P*] where *P* = (*F*_o_^2^ + 2*F*_c_^2^)/3
1231 reflections	(Δ/σ)_max_ < 0.001
102 parameters	Δρ_max_ = 0.31 e Å^−^^3^
0 restraints	Δρ_min_ = −0.29 e Å^−^^3^

### Special details

**Table:**

Geometry. All e.s.d.'s (except the e.s.d. in the dihedral angle between two l.s. planes) are estimated using the full covariance matrix. The cell e.s.d.'s are taken into account individually in the estimation of e.s.d.'s in distances, angles and torsion angles; correlations between e.s.d.'s in cell parameters are only used when they are defined by crystal symmetry. An approximate (isotropic) treatment of cell e.s.d.'s is used for estimating e.s.d.'s involving l.s. planes.
Refinement. Refinement of *F*^2^ against ALL reflections. The weighted *R*-factor *wR* and goodness of fit *S* are based on *F*^2^, conventional *R*-factors *R* are based on *F*, with *F* set to zero for negative *F*^2^. The threshold expression of *F*^2^ > σ(*F*^2^) is used only for calculating *R*-factors(gt) *etc*. and is not relevant to the choice of reflections for refinement. *R*-factors based on *F*^2^ are statistically about twice as large as those based on *F*, and *R*- factors based on ALL data will be even larger.

### Fractional atomic coordinates and isotropic or equivalent isotropic displacement parameters (Å^2^)

**Table:**

	*x*	*y*	*z*	*U*_iso_*/*U*_eq_	
Ni1	0.0000	0.69428 (4)	0.2500	0.02321 (14)	
N1	0.10680 (18)	0.7919 (2)	0.49121 (17)	0.0423 (6)	
H1A	0.1428	0.7976	0.4363	0.051*	
H1B	0.1341	0.8297	0.5496	0.051*	
N2	−0.04038 (14)	0.6630 (2)	0.40062 (14)	0.0264 (4)	
O1	−0.16405 (12)	0.68137 (17)	0.20264 (12)	0.0314 (4)	
O2	−0.33123 (12)	0.5835 (2)	0.19153 (12)	0.0369 (4)	
O3	0.0000	0.9133 (2)	0.2500	0.0401 (6)	
H3	0.0570	0.9433	0.2270	0.060*	
C1	−0.23701 (16)	0.5998 (2)	0.23761 (16)	0.0254 (5)	
C2	−0.20703 (19)	0.5149 (3)	0.33761 (18)	0.0336 (5)	
H2A	−0.2751	0.4820	0.3650	0.040*	
H2B	−0.1657	0.4283	0.3204	0.040*	
C3	0.00918 (19)	0.7248 (3)	0.48595 (17)	0.0295 (5)	
C4	−0.14006 (17)	0.5971 (2)	0.42235 (17)	0.0280 (5)	
C5	−0.16604 (19)	0.6117 (3)	0.52138 (18)	0.0364 (6)	
H5	−0.2299	0.5739	0.5468	0.044*	
S1	−0.06560 (5)	0.71004 (8)	0.59589 (5)	0.03934 (19)	

### Atomic displacement parameters (Å^2^)

**Table:**

	*U*^11^	*U*^22^	*U*^33^	*U*^12^	*U*^13^	*U*^23^
Ni1	0.0186 (2)	0.0286 (2)	0.0224 (2)	0.000	0.00139 (15)	0.000
N1	0.0395 (12)	0.0586 (15)	0.0285 (11)	−0.0167 (11)	0.0017 (9)	−0.0117 (10)
N2	0.0241 (9)	0.0319 (10)	0.0229 (9)	−0.0007 (8)	0.0009 (7)	−0.0001 (8)
O1	0.0225 (8)	0.0422 (9)	0.0291 (9)	−0.0047 (7)	0.0005 (6)	0.0090 (7)
O2	0.0217 (8)	0.0551 (11)	0.0327 (9)	−0.0077 (7)	−0.0033 (6)	0.0127 (8)
O3	0.0262 (12)	0.0300 (12)	0.0666 (17)	0.000	0.0190 (12)	0.000
C1	0.0217 (11)	0.0292 (11)	0.0252 (11)	0.0004 (9)	0.0016 (9)	−0.0006 (9)
C2	0.0290 (11)	0.0352 (13)	0.0354 (13)	−0.0065 (10)	−0.0038 (10)	0.0099 (11)
C3	0.0294 (12)	0.0343 (13)	0.0246 (12)	0.0031 (10)	0.0014 (9)	−0.0006 (10)
C4	0.0239 (10)	0.0314 (12)	0.0281 (12)	0.0023 (9)	−0.0004 (9)	0.0071 (10)
C5	0.0278 (12)	0.0504 (15)	0.0311 (13)	0.0004 (11)	0.0039 (10)	0.0096 (12)
S1	0.0384 (4)	0.0570 (4)	0.0230 (3)	0.0039 (3)	0.0044 (3)	−0.0030 (3)

### Geometric parameters (Å, °)

**Table:**

Ni1—O1	2.0243 (15)	O2—C1	1.243 (3)
Ni1—O1^i^	2.0243 (15)	O3—H3	0.8200
Ni1—O3	1.999 (2)	C1—C2	1.510 (3)
Ni1—N2	2.0465 (18)	C2—C4	1.494 (3)
Ni1—N2^i^	2.0465 (18)	C2—H2A	0.9700
N1—C3	1.326 (3)	C2—H2B	0.9700
N1—H1A	0.8600	C3—S1	1.742 (2)
N1—H1B	0.8600	C4—C5	1.337 (3)
N2—C3	1.322 (3)	C5—S1	1.726 (3)
N2—C4	1.397 (3)	C5—H5	0.9300
O1—C1	1.266 (3)		
			
O3—Ni1—O1	93.34 (5)	O2—C1—C2	118.64 (19)
O3—Ni1—O1^i^	93.34 (5)	O1—C1—C2	118.57 (18)
O1—Ni1—O1^i^	173.33 (9)	C4—C2—C1	115.36 (19)
O3—Ni1—N2	98.02 (5)	C4—C2—H2A	108.4
O1—Ni1—N2	87.90 (7)	C1—C2—H2A	108.4
O1^i^—Ni1—N2	91.17 (7)	C4—C2—H2B	108.4
O3—Ni1—N2^i^	98.02 (5)	C1—C2—H2B	108.4
O1—Ni1—N2^i^	91.17 (7)	H2A—C2—H2B	107.5
O1^i^—Ni1—N2^i^	87.90 (7)	N2—C3—N1	125.1 (2)
N2—Ni1—N2^i^	163.96 (11)	N2—C3—S1	113.70 (17)
C3—N1—H1A	120.0	N1—C3—S1	121.23 (17)
C3—N1—H1B	120.0	C5—C4—N2	115.1 (2)
H1A—N1—H1B	120.0	C5—C4—C2	125.3 (2)
C3—N2—C4	110.83 (19)	N2—C4—C2	119.58 (19)
C3—N2—Ni1	125.93 (16)	C4—C5—S1	111.14 (18)
C4—N2—Ni1	121.96 (14)	C4—C5—H5	124.4
C1—O1—Ni1	128.58 (14)	S1—C5—H5	124.4
Ni1—O3—H3	109.5	C5—S1—C3	89.19 (11)
O2—C1—O1	122.8 (2)		
			
O3—Ni1—N2—C3	−50.65 (19)	C4—N2—C3—N1	177.7 (2)
O1—Ni1—N2—C3	−143.73 (19)	Ni1—N2—C3—N1	−15.1 (3)
O1^i^—Ni1—N2—C3	42.88 (19)	C4—N2—C3—S1	−1.8 (2)
N2^i^—Ni1—N2—C3	129.35 (19)	Ni1—N2—C3—S1	165.31 (11)
O3—Ni1—N2—C4	115.15 (16)	C3—N2—C4—C5	1.4 (3)
O1—Ni1—N2—C4	22.08 (17)	Ni1—N2—C4—C5	−166.37 (17)
O1^i^—Ni1—N2—C4	−151.32 (17)	C3—N2—C4—C2	−177.3 (2)
N2^i^—Ni1—N2—C4	−64.85 (16)	Ni1—N2—C4—C2	15.0 (3)
O3—Ni1—O1—C1	−135.01 (18)	C1—C2—C4—C5	126.8 (3)
N2—Ni1—O1—C1	−37.09 (19)	C1—C2—C4—N2	−54.7 (3)
N2^i^—Ni1—O1—C1	126.88 (19)	N2—C4—C5—S1	−0.3 (3)
Ni1—O1—C1—O2	−167.76 (16)	C2—C4—C5—S1	178.27 (18)
Ni1—O1—C1—C2	10.2 (3)	C4—C5—S1—C3	−0.6 (2)
O2—C1—C2—C4	−140.5 (2)	N2—C3—S1—C5	1.44 (19)
O1—C1—C2—C4	41.5 (3)	N1—C3—S1—C5	−178.2 (2)

Symmetry codes: (i) −*x*, *y*, −*z*+1/2.

### Hydrogen-bond geometry (Å, °)

**Table:**

*D*—H···*A*	*D*—H	H···*A*	*D*···*A*	*D*—H···*A*
N1—H1A···O1^i^	0.86	2.10	2.816 (3)	140
N1—H1B···O2^ii^	0.86	1.99	2.839 (3)	170
O3—H3···O2^iii^	0.82	1.94	2.7211 (19)	158

Symmetry codes: (i) −*x*, *y*, −*z*+1/2; (ii) *x*+1/2, −*y*+3/2, *z*+1/2; (iii) *x*+1/2, *y*+1/2, *z*.
